# Draft Genome Sequence of the Oomycete Globisporangium splendens Strain rgcb-1

**DOI:** 10.1128/MRA.01006-19

**Published:** 2020-04-16

**Authors:** Rani Jalajakumari Reghu, Biju Vadakkemukadiyil Chellappan, Shenu Hudson Beena, Anu Sasi, Soniya Eppurathu Vasudevan, Achuthsankar Sukumaran Nair

**Affiliations:** a Department of Computational Biology and Bioinformatics, University of Kerala, Kariavattom Campus, Trivandrum, Kerala, India; b Rajiv Gandhi Centre for Biotechnology, Trivandrum, Kerala, India; University of California, Riverside

## Abstract

Globisporangium splendens (formerly Pythium splendens) is an oomycete pathogen of many economically important vegetable crops. Here, we present the first draft genome of P. splendens, which comprises 197 scaffolds with a total length of 53.3 Mb and 17,350 predicted protein-coding genes.

## ANNOUNCEMENT

*Pythium* is a genus of oomycetes, comprising over 355 species with a worldwide distribution (1). Many *Pythium* species are destructive pathogens, especially of monocotyledonous plants, whereas some species can cause diseases in fish, red algae, and mammals, including humans. Globisporangium splendens (formerly Pythium splendens) is an important plant pathogen that causes rots of stems and roots and pre- and postemergence seedling damping off in many plants, including (but not limited to) black pepper, maize, muskmelon, ginger, and oil palm (2–6). Although the whole genomes of many *Pythium* species are available, the whole genome of Pythium splendens is completely absent, which limits the understanding of its virulence mechanism. Here, we present the first draft genome sequence of P. splendens, which infects black pepper from Kerala, India. We hope that this genome sequence will be a valuable resource for gaining a better understanding of the mechanism underlying the pathogenicity of this destructive pathogen.

P. splendens culture was obtained from Kerala Agricultural University (Thiruvananthapuram, India). The isolate was cultured on potato-dextrose agar plates, and DNA was extracted from mycelia using the DNeasy plant minikit (Qiagen). Illumina paired-end (69 million reads) and PacBio single-molecule real-time (SMRT) (0.7 million reads) libraries were sequenced using the Illumina HiSeq 2500 and PacBio Sequel platforms, respectively, according to the instruction manual for each platform. Cutadapt (v2.3) was used with default parameters to remove adapter sequences, primers, poly(A) tails, and low-quality sequences (7). In total, 6.9 Gb and 10.2 Gb of Illumina and PacBio data, respectively, were obtained. MaSuRCA (v2.2.1) was used to build a hybrid assembly using both Illumina and PacBio data (8). The total length of the final assembly was 53.3 Mb, distributed in 198 scaffolds with a maximum scaffold length of 1.7 Mb. QUAST was used to assess the assembly scaffold quality (*N*_50_, 342.1 kb; *N*_75_, 237.8 kb; *L*_50_, 44; *L*_75_, 77; longest scaffold, 1.7 Mb; number of scaffolds of >50 kb, 180; number of scaffolds of >10 kb, 195) (9). The total GC content of the assembly was 52.6%. The Benchmarking Universal Single-Copy Orthologs (BUSCO) (v3.0.1) program identified 201 of 215 conserved protist genes from protists_ensembl (http://busco.ezlab.org), reflecting the fact that the P. splendens draft genome is 93.5% complete. A combination of *de novo* and homology-based approaches was used to mask 40% of the repetitive sequences in the genome. The protein-coding genes were predicted with the AUGUSTUS program implemented in Blast2Go (v5.1.13) software, using the previously sequenced transcriptome data of P. splendens as hints, with the following parameters: map threshold, 30; minimum read alignment, 11; minimum intron length, 32; minimum exon length, 300; depth coverage, 20; this resulted in the prediction of 17,350 protein-coding genes (10, 11). Functional annotation using Blast2Go (v5.1.13) software revealed that 10,145 proteins had homologs in the NCBI databases. SECRETOOL analysis predicted 713 secretory proteins, of which 88 were found to possess signatures corresponding to RXLR effectors (12, 13) and 1,760 had Pfam domains (14).

### Data availability.

Both Illumina and PacBio raw reads have been submitted to the NCBI SRA under BioProject accession number PRJNA548776. The whole-genome shotgun project and annotation file have been deposited in DDBJ/ENA/GenBank under accession number VFIW00000000 (BioSample accession number SAMN12049464).
